# ﻿*Agapetes lichengii* (Ericaceae), a new species from Xizang, China

**DOI:** 10.3897/phytokeys.269.177341

**Published:** 2026-01-15

**Authors:** Yi-Hua Tong, Xue-He Ye, Jing-Bo Ni, Bing-Mou Wang, Xi-Rong Zheng

**Affiliations:** 1 State Key Laboratory of Plant Diversity and Specialty Crops & Laboratory of Plant Resources Conservation and Sustainable Utilization, South China Botanical Garden, Chinese Academy of Sciences, Guangzhou, Guangdong, 510650, China; 2 South China National Botanical Garden, Chinese Academy of Sciences, Guangzhou, Guangdong, 510650, China; 3 Panyu Central Hospital, Guangzhou, Guangdong, 511402, China; 4 Guangzhou Institute of Forestry and Landscape Architecture, Guangzhou, Guangdong, 510540, China

**Keywords:** *
Agapetes
*, Mêdog, morphology, stigma ornamentation, taxonomy

## Abstract

A new species of Ericaceae, *Agapetes lichengii*, from Xizang Autonomous Region, China, is described and illustrated. The new species is morphologically most similar to *A. pentastigma*, but differs by having leaf blade with an obtuse or slightly auriculate leaf base, corolla with a constricted basal part and V-shaped stripes, spurless anthers and stigma without obvious gap between crenae. Detailed description, colour plates, and taxonomic notes on the new species are provided.

## ﻿Introduction

The genus *Agapetes* D. Don ex G. Don is a relatively large genus of the tribe Vaccinieae in Ericaceae, with about 115 species mainly distributed from the eastern Himalayas through southwest China and Indochina to southeast Asia (Fang 1991, Fang and Stevens 2005, POWO 2025). In China, 67 species of *Agapetes* have been recorded up to now including the newly described *A. hongheensis* Y.H.Tong & C.Y.Zou, *A. brevituba* X.C.Xie & Y.H.Tan, *A. wenpeiana* Bin Yang & Y.H.Tan and *A. mingyuaniana* H.B.Ding, Bin Yang & Y.H.Tan (Yang et al. 2023, 2024, 2025; Tong et al. 2024; Zou et al. 2025; Xie et al. 2025).

During a field trip to Mêdog (Motuo) County, Xizang Autonomous Region, China in January 2024, we encountered an interesting *Agapetes* species bearing young fruits. Some living plants were collected and cultivated in a greenhouse in Guangzhou City, Guangdong Province, China, which is owned by the fourth author. In June 2025, this species blooms with beautiful flowers. Its greenish yellow corolla with crimson bands immediately reminded us of *A. pentastigma* J.Murata, Nob. Tanaka & H.Murata (Tanaka et al. 2016), a species reported from Myanmar. However, some detailed characteristics of leaves, corolla, stamens and pistil are different. After a detailed comparison of this species and similar species from China and neighbouring countries (King and Gamble 1910; Dop 1930; Airy Shaw 1948, 1958, 1960a, 1960b, 1968; Sleumer 1967; Pham 1999; Nguyen 2005; Fang and Stevens 2005; Watthana 2015; Tanaka et al. 2016, Murata and Murata 2020; Tong et al. 2022), we concluded that this species is new to science, as described and illustrated below.

## ﻿Materials and methods

Measurements and descriptions of this new species were based on both living plants and dried specimens collected from the wild plants in Mêdog, Xizang and the cultivated plants in Guangzhou, Guangdong. Measurements were performed with a ruler and small plant parts were observed and measured under a stereoscope (Mshot-MZ101, Guangzhou Micro-shot Technology Co., Ltd, Guangzhou, China). The terminology followed Beentje (2016). Acronyms of herbaria follow the Index Herbariorum (Thiers 2025).

## ﻿Taxonomic treatment

### ﻿ Agapetes lichengii

Taxon classificationPlantaeEricalesEricaceae

Y.H.Tong & B.M.Wang sp. nov.

AE584D2B-9169-531F-B153-D295D2F5AF09

urn:lsid:ipni.org:names:77375067-1

Figs 1, 2

#### Type.

China. Xizang Autonomous Region • Mêdog County, Beibeng Xiang, Deyanggou, ca. 680 m a.s.l.; 15 January 2024; *Jing-Bo Ni, Bing-Mou Wang & Cheng Li TYH-2813* (holotype IBSC, isotypes IBSC, PE).

**Figure 1.**
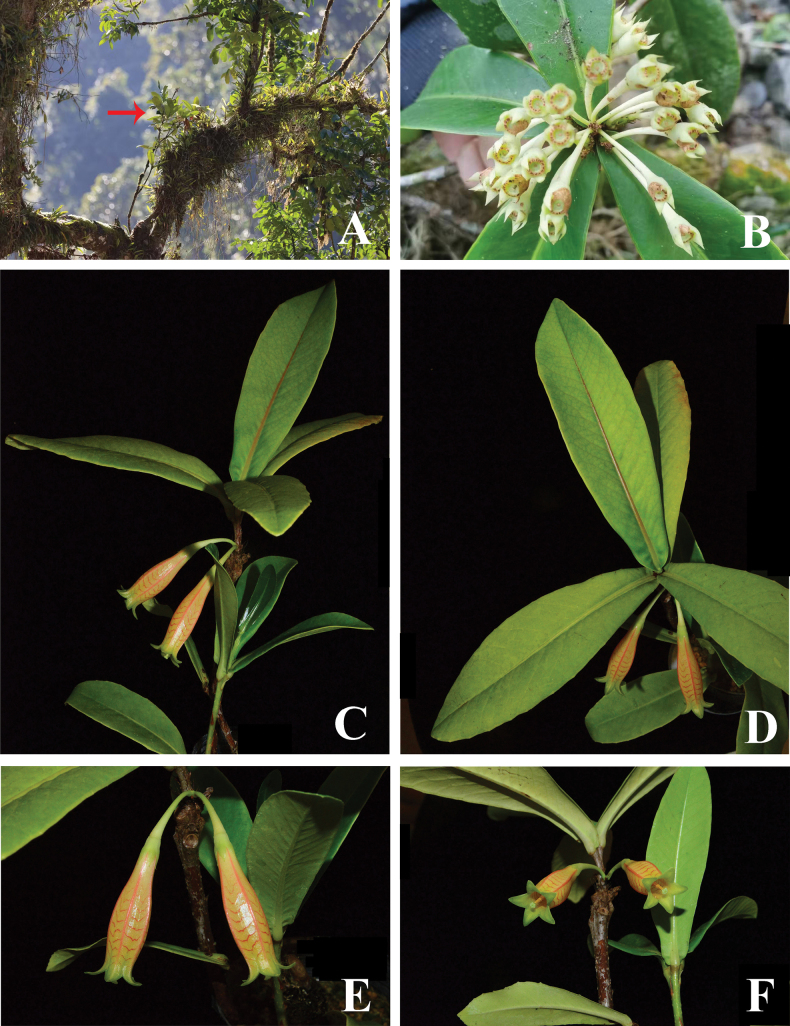
*Agapetes lichengii***A.** Habit, the arrow shows a plant of this species epiphytic on a big tree trunk; **B.** Infructescence; **C.** Flowering branch and leaf branch; **D.** Leaves; **E.** Lateral view of flowers, showing the V-shaped transverse bands on corolla tube; **F.** Front view of flowers. Photos (**A, B**) by Cheng Li, (**C–F**) by Y.H. Tong. (**A.** Unvouchered; **B.** based on *J.B. Ni et al. TYH-2813*; **C–F.** based on *B.M. Wang s.n.*).

**Figure 2.**
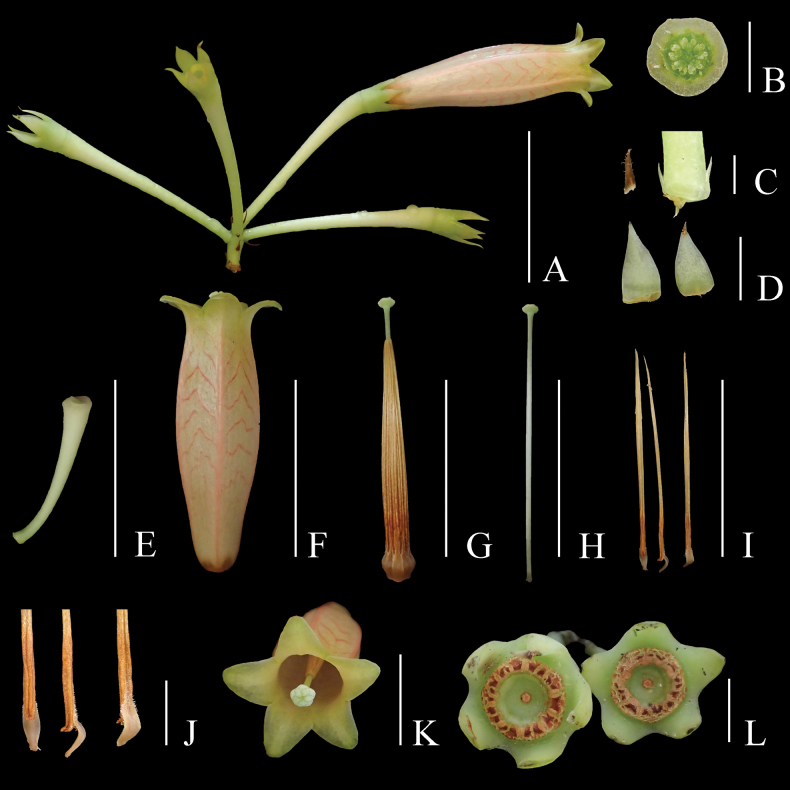
*Agapetes lichengii*. **A.** Inflorescence; **B.** Ovary, transection view; **C.** Bract and bracteoles; **D.** Calyx lobes, adaxial view; **E.** Pedicel; **F.** Corolla; **G.** Style and androecium; **H.** Style; **I.** Stamens, adaxial (left), lateral (middle) and abaxial (right) view; **J.** Lower part of stamens, showing pubescent filaments and echinate thecae, adaxial (left), lateral (middle) and abaxial (right) view; **K.** Front view of flower, showing the 5-crenated capitate stigma and corolla lobes; **L.** Front view of young fruits, showing the rounded ring of 10 distinct filament scars. Scale bars: 3 cm (**A, E, F, G, H, I**); 5 mm (**B, D, J, L**); 2 mm (**C**); 1 cm (**K**).

#### Diagnosis.

**Figure 3.**
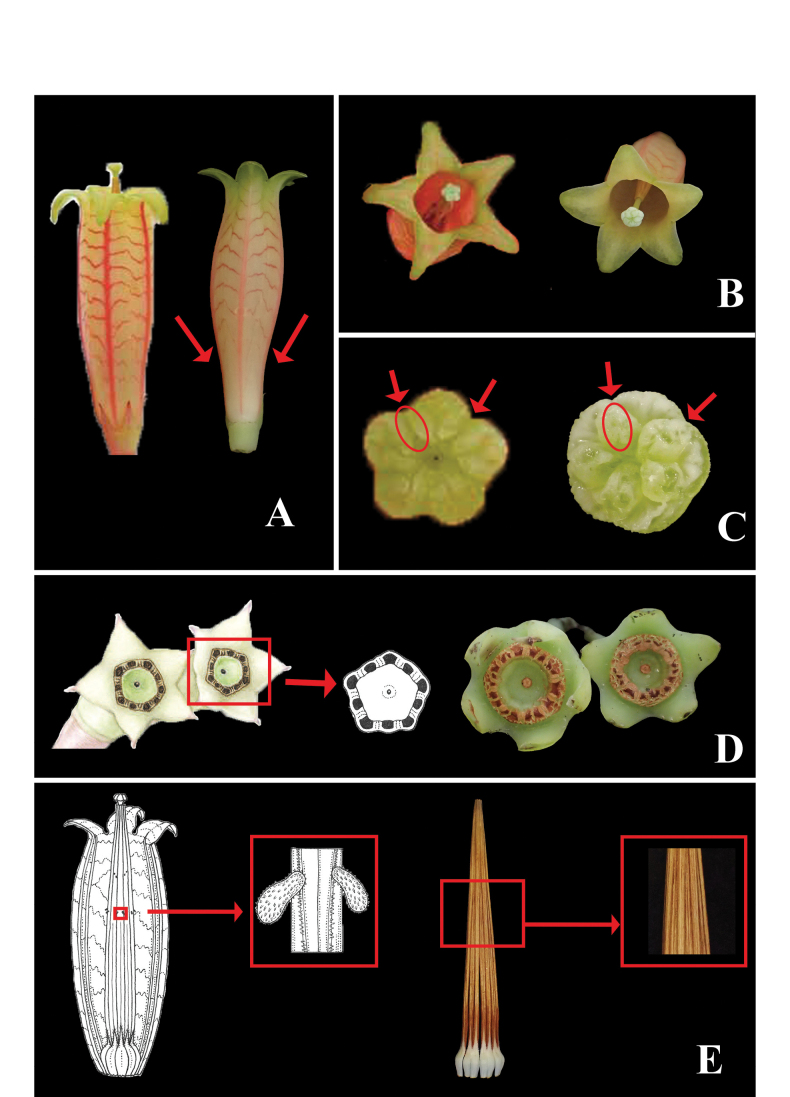
Morphological comparison between *Agapetes pentastigma* (left) and *A. lichengii* (right). **A.** Corollas, showing different corolla shapes, bending degree of corolla lobes and transverse bands; **B.** Front view of flowers, showing different shapes of corolla lobes; **C.** Close-up view of stigma, showing the gap between stigma crenae present in *A. pentastigma* and absent in *A. lichengii*; **D.** Top view of fruits, showing the pentagonal (left) and rounded (right) filament scars; **E.** Androecium, showing the spurs on the tubules present in *A. pentastigma* and absent in *A. lichengii*. Illustrations or photos of *A. pentastigma* are from Tanaka et al. (2016) and Murata and Murata (2020).

*Agapetes lichengii* is morphologically similar to *A. pentastigma* in having pseudo-whorled leaves, corymbose inflorescences, greenish yellow corolla with crimson transverse bands and capitate stigmas, but can be distinguished from it by the elliptic to oblanceolate (vs. oblong-lanceolate) leaf blades with an obtuse or slightly auriculate (vs. attenuate) base, corolla with a constricted(vs. not constricted) basal part, V-shaped (vs. ladder-like) stripes and triangular and spreading or slightly reflexed (vs. triangular-lanceolate and reflexed) lobes, anthers without dorsal spurs (vs. with two small dorsal spurs), stigma without gap between crenae (vs. with obvious gap between crenae), and filament scars forming a rounded ring (vs. a pentagonal ring) on fruit top (Fig. 3; Table 1).

**Table 1.** Morphological comparison of *Agapetes lichengii* and *A. pentastigma*.

Characters	*A. lichengii*	*A. pentastigma*
Leaf blade base	Obtuse or slightly auriculate	Attenuate
Basal part of corolla	Constricted	Not constricted
Stripes on corolla	V-shaped	Ladder-like
Corolla lobes	Triangular, spreading or slightly reflexed	Triangular-lanceolate, reflexed
Spurs on the back of anther tubules	Absent	Present
Gap between stigma crenae	Absent	Present
Filament scars on fruit top	A rounded ring	A pentagonal ring

#### Description.

Evergreen shrubs, epiphytic, with woody fusiformed tubers. Branches robust, dark brown, glabrous. Leaves alternate, pseudo-whorled; petiole subsessile, 1–3 mm long, light green, glabrous; blades leathery, adaxially green, abaxially pale green, elliptic to oblanceolate, 13–22 × 4.2–7.5 cm, length: width 2.6–4.2, glabrous on both sides, midvein impressed at base, flat or slightly elevated on the upper half, raised abaxially, lateral veins 17–19 pairs, together with veinlets raised and conspicuous on both sides; leaf base obtuse or slightly auriculate, margin repand, subentire or obtusely dentate, each dentation with a gland at the tip, apex obtuse or acute. Inflorescence corymbose, 2–9-flowered, axillary; rachis 2–9 mm long, glabrous; bracts small, lanceolate to linear, 2–4 mm long, margin ciliate, apex acute; bracteoles 2, inserted at the base of pedicel, lanceolate, glabrous, 1–2 mm long; pedicels expanded upwards, clavate, 2.6–3.5 cm long, glabrous, articulated with calyx. Calyx tube green, 4–5 mm long, glabrous, lobes narrowly triangular, 7–15 × ca. 4 mm, glabrous, apex acute. Corolla pale yellow to greenish yellow, cerise on angles, with crimson V-shaped transverse bands between angles, tubular, constricted at base, slightly wider in the middle part, 5-angled; tube 4.5–5 × ca. 0.8 cm, glabrous on both sides; lobes yellowish green, triangular, 8–9 × 6–7 mm, apex acute, glabrous, spreading or slightly reflexed. Stamens 10, 4.5–4.7 cm long, filaments flat, curved, 5–6 mm long, pubescent on the upper part; anthers coherent, 4.1–4.4 cm long, thecae densely echinate, brown, 6–7 mm long, tubules 3.5–3.7 cm long, sparsely echinate, opening by apical pores, without spurs on the back. Ovary inferior, pseudo-10-locular, each locule with several ovules; disc yellowish, glabrous; style slender, ca. 5 cm long, glabrous, slightly expanded upwards, exserted ca. 6 mm from the connate anther tubules; stigma capitate, 5-crenated, without obvious gap between crenae. Infructescence rachis 1.9–2 cm long, ca. 4 mm thick, glabrous. Fruit pedicel greenish white, 2.8–3 cm long, expanded upwards, ca. 6 mm thick at top. Young fruits greenish, cup-shaped, 1.5–1.7 cm long including persistent calyx lobes, 0.8–0.9 cm in diam., flat topped with a rounded ring of 10 distinct filament scars.

#### Etymology.

The species is named in honor of Mr. Li Cheng, who devoted lots of time and energy to the biodiversity conservation of Mêdog County, and is also one of the discoverers of this new species. The Chinese name is given as 李成树萝卜 (pinyin: lĬ chéng shù luó bo).

#### Phenology.

Flowering from October to November; fruiting in March.

#### Distribution and habitat.

At present, *Agapetes lichengii* is known only from the type locality, i.e., Mêdog County, Xizang Autonomous Region, China. It usually grows on large tree trunks near the crown in rainforests at an elevation of ca. 680 m.

#### Additional specimen examined.

*Agapetes lichengii*: China. Xizang Autonomous Region • Medog County, Beibeng Xiang, Deyanggou, ca. 680 m a.s.l., cultivated in a greenhouse in Guangzhou; 5 June 2025; *Bing-Mou Wang s.n.* (paratype IBSC).

*Agapetes pentastigma*: Myanmar. Kachin State • Along the Ledo Road, between Namyung and Shinbweyan, 5–7 miles from Shinbweyan toward Namyung, in border area of Sagaing Region, Hukaung Valley Tiger Reserve, 26°42'32"N, 96°11'55"–13'01"E, cultivated in the Setsunan University; 11 November 2014, *Hiroko Murata & Jin Murata 1402* (holotype TI, not seen; isotypes NY, image!, RAF, not seen, TNS, image!).

#### Taxonomic notes.

According to Airy Shaw’s infrageneric classification (Airy Shaw 1935, 1948, 1958), *A. lichengii* should be assigned to Agapetes subser. Coriaceae Airy Shaw due to its large leathery leaf blades with a repand margin, corymbose inflorescence and large flowers. *Agapetes lichengii* is morphologically mostly similar to *A. pentastigma*, but these two species are different in the morphology of leaves, corolla, anthers, stigma and fruit. Detailed morphological comparison is presented in the diagnosis part. It should be noted that some characters may change during different development stages, such as the shape of stripes on corolla tube, the bend degree of corolla lobes or the shape of stamen scar, so we made the comparison by using materials nearly at the same stage, such as fully open flowers or mature fruits, and we found that these characters are very useful to distinguish these two species. As for the differences in corolla tube shape and stigma morphology, they are actually consistent and irrelevant to the development stage. Besides, the two species are allopatric: *A. lichengii* is only found in Mêdog County, while *A. pentastigma* is endemic to Kachin State of Myanmar.

There are two kinds of stigma in *Agapetes* species: (1) small, truncate (or punctate) and inconspicuous; (2) capitate and conspicuous (Airy Shaw 1935, 1948, 1958; Fang and Stevens 2005; Murata and Murata 2020). While the ‘small’ stigma is prevalent across a large portion of the genus, only a few taxa (e.g., *A. affinis* (Griff.) Airy Shaw, *A. bhareliana* (Airy Shaw) D.Banik & Sanjappa, *A. hillii* Brandis, *A. loranthiflora* D.Don ex G.Don (fide Watthana 2015), *A. marginata* Dunn, *A. odontocera* (Wight) Hook. f., *A. pentastigma*, *A. salicifolia* C.B.Clarke, *A. setigera* D.Don ex G.Don, *A. sikkimensis* Airy Shaw, *A. variegata* D.Don ex G.Don, and the present new species), possess the ‘capitate’ stigma (Banik and Sanjappa 2014; Murata and Murata 2020). Consequently, stigma morphology is seldom used for species delimitation. The two similar *Agapetes* species (i.e. *A. lichengii* and *A. pentastigma*) presented here both have ‘capitate’ stigma, but the detailed ornamentations on the stigma surface are very different (Fig. 3C). And this distinguishing character is so stable and consistent that we believe that it could be served as a useful character for identification of *Agapetes* species. But this character seems to be only available for living material, and the stigma surface ornamentation would become barely seen when dried due to the shrinkage. As most descriptions of stigma in *Agapetes* are oversimplified, and some taxa even lack descriptions of their stigmas, we suggest that researchers pay more attention to the stigma surface ornamentation of *Agapetes* species in the future, which might offer us better understanding of the species of this genus.

## Supplementary Material

XML Treatment for Agapetes lichengii

## ﻿Additional information

### Use of AI

No use of AI was reported.

### Author ORCIDs

Yi-Hua Tong https://orcid.org/0000-0002-5034-005X

Xue-He Ye https://orcid.org/0000-0001-5718-5737

Bing-Mou Wang https://orcid.org/0000-0002-1103-2599

Xi-Rong Zheng https://orcid.org/0000-0002-5174-2552

### Data availability

All of the data that support the findings of this study are available in the main text.
